# Irradiation-Induced Intestinal Damage Is Recovered by the Indigenous Gut Bacteria *Lactobacillus acidophilus*

**DOI:** 10.3389/fcimb.2020.00415

**Published:** 2020-08-18

**Authors:** Panida Sittipo, Huy Quang Pham, Chang Eon Park, Gi-Ung Kang, Yong Zhi, Hyun Jung Ji, Ayeung Jang, Ho Seong Seo, Jae-Ho Shin, Yun Kyung Lee

**Affiliations:** ^1^Department of Integrated Biomedical Science, Soonchunhyang Institute of Medi-Bio Science, Soonchunhyang University, Cheonan, South Korea; ^2^Department of Applied Biosciences, Kyungpook National University, Daegu, South Korea; ^3^Radiation Biotechnology Research Division, Advanced Radiation Technology Institute, Korea Atomic Energy Research Institute, Jeongeup, South Korea

**Keywords:** gamma irradiation, intestinal microbiota, intestinal epithelial cells, intestinal organoids, *Lactobacillus acidophilus*

## Abstract

The intestinal tract is one of the most sensitive organs following irradiation. The protective effect of specific indigenous microbiota on irradiation-induced damage to intestinal epithelial cells has not been reported. Mice were irradiated with a single dose of 6 Gy of gamma rays. The intestinal damage was analyzed by histopathology. Intestinal stemness and differentiation were determined by intestinal organoid culture. Microbiota community was observed by high-throughput 16S rRNA gene sequencing and oligotyping analysis. We showed that distal small intestine was damaged by sublethal dose of gamma irradiation. Intestinal organoids derived from the irradiated mice showed defects in budding and mucin expression, suggesting the detrimental effect of irradiation on the intestinal stemness and differentiation. In addition, irradiation reduced intestinal immunoglobulin A level, concomitant with decreased microbiota diversity based on our high-throughput 16S rRNA gene sequencing data. Especially, the relative abundance of *Lactobacillus* was reduced at early time point post-irradiation; however, it was recovered at late time point. Oligotyping analysis within the *Lactobacillus* genus indicated that *Lactobacillus*-related oligotype 1 (OT1) including *Lactobacillus acidophilus* might drive recovery after irradiation as it was associated with increased long-term numbers post-exposure. We showed that treatment with heat-killed *L. acidophilus* rescued the budding-impaired organoids and induced sufficient differentiation in epithelial cells, and particularly mucin-producing cells, in intestinal organoids. This study provides the first evidence that the indigenous gut bacteria *L. acidophilus* enhance intestinal epithelial function with respect to irradiation-induced intestinal damage by improving intestinal stem cell function and cell differentiation.

## Introduction

Gamma radiation is widely used for clinical applications (Deacon et al., 2008; De Andrade et al., 2014). Sublethal doses (6–10 Gy) of gamma irradiation have been used effectively for bone marrow transplantation, immunosuppression, and cancer therapy. Although it is a valuable therapeutic tool, it is well-known to cause serious damage to the body. The gastrointestinal tract is one of the most radio-sensitive organs following exposure to sublethal doses of gamma irradiation (Hauer-Jensen et al., 2007; Donnelly et al., 2010; Ki et al., 2014). Irradiation significantly affects the intestinal epithelial cells (IECs) and gut microbiota of the mucosal intestinal system (Atasoy et al., 2010; Kim et al., 2015; Du et al., 2018). The intestinal microbiota has an impact on host physiology, whereas the host in turn shapes the intestinal microbial community (Sekirov et al., 2010; Peck et al., 2017; Contijoch et al., 2019). Host cells, and specifically IECs, can recognize and respond to signals generated by the intestinal microbiota or associated molecules, resulting in a reinforced intestinal barrier function and the maintenance of intestinal homeostasis (Round and Mazmanian, 2009; Peterson and Artis, 2014; Nigro and Sansonetti, 2015; Pan et al., 2018). IECs not only form the barrier but also function differently based on specific cell types. IECs are classified into different specific functional cell types including intestinal stem cells (ISCs), Paneth cells (lysozyme-producing cells), which are located in the intestinal crypts, goblet cells (mucus-secreting cells), enteroendocrine cells, and enterocytes (absorptive cells), which are localized in the intestinal villi (Fair et al., 2018).

The intestinal microbiota influences the proliferation and differentiation of IECs, as evidenced by abnormalities in the intestinal tract of germ-free or antibiotic-treated animal models (Khoury et al., 1969a,b). Thus, changes in the composition of the intestinal microbiota following irradiation might affect intestinal development and self-renewal, which could lead to severe radiation-induced intestinal toxicity (Kim et al., 2015; Wang et al., 2015; Cui et al., 2017; Peck et al., 2017). Accordingly, reconstitution of the original microbiota by fecal microbiota transplantation and the administration of probiotics are known to protect against radiation-induced intestinal toxicity by improving intestinal integrity and inducing anti-inflammatory processes (Demirer et al., 2006; Ciorba and Stenson, 2009; Ciorba et al., 2012; Cui et al., 2017). However, direct evidence showing the protective effect of specific indigenous microbiota on irradiation-induced damage to IECs, and specifically to improve cell characteristics including stemness and detailed cell differentiation, has not been reported.

Herein, we observed that the distal small intestine was the most radio-sensitive component, among parts of the intestine, in total body gamma-irradiated mice, as determined by the damaged villi–crypt structure, shortened villus length, and reduced expression of stem cell and proliferative genes. Consistently, intestinal organoids derived from the irradiated mice showed a loss of budding and reduced mucin production, suggesting that sublethal doses of gamma irradiation suppress the stemness and cell differentiation of IECs in the distal small intestine. In addition, we found that the relative abundance of bacteria of the genus *Lactobacillus* was dramatically reduced immediately after irradiation and was recovered long-term post-irradiation. Oligotyping analysis indicated that *Lactobacillus*-related oligotype 1 (OT1) including *Lactobacillus acidophilus* was indicative of this changing pattern as it was associated with increased numbers post-exposure, suggesting that *L. acidophilus* might play a role in the recovery of radiation-induced intestinal damage. Consistently, treatment with heat-killed *L. acidophilus* was found to rescue the damage to epithelial cells by inducing a budding organoid phenotype and mucin-producing cells in intestinal organoids. Our data suggest the protective effects of the indigenous bacteria *L. acidophilus* against epithelial damage during radiation-induced intestinal toxicity.

## Materials and Methods

### Mice and Irradiation Exposure

Eight-week-old C57BL/6L male mice were purchased from ORIENT Bio Korea (Korea) and were maintained in the specific pathogen-free animal facility at the Korea Atomic Energy Research Institute (KAERI). Mice were irradiated with a single dose of 6 Gy of gamma rays using a gammacell 40 Exactor (Atomic Energy of Canada Limited, Canada). All procedures were performed according to guidelines of the Institutional Animal Care and Use Committee at the KAERI. All procedures were performed according to guidelines of the Institutional Animal Care and Use Committee at the Korea Atomic Energy Research Institute.

### *L. acidophilus* Culture and Heat-Killed Bacteria Preparation

*L. acidophilus* (no. 3171) was purchased from the Korean Collection for Type Cultures (KCTC, Korea), Biological Resource Center (BRC) and were grown anaerobically at 37°C in de Man, Rogosa, Sharpe (MRS) broth (BD Difco, Sparks, MD). For heat-killed bacteria, cultured *L. acidophilus* were washed twice with PBS and heated in 1 ml PBS at 100°C for 30 min.

### Stool Sample Collection and Next Generation Sequencing

Stool samples were collected 1 day before irradiation (D−1) and on three different days post-irradiation (D1, D3, and D10). Bacterial genomic DNA was extracted from the stool samples using PowerFecal^®^ kits (Mo Bio Laboratories Inc., Carlsbad, CA) following the instructions of the manufacturer. For library preparation, primers targeting the highly variable V4–V5 region of the 16S rRNA gene (forward, GTGCCAGCMGCCGCGGTAA; reverse, CCGTCAATTCMTTTRAGTTT) with adaptors were used. Then, the pooled library DNA was quantified using a Qubit 2.0 Fluorometer (Thermo Fisher Scientific, Waltham, MA) and verified with the 2100 Bioanalyzer (Agilent Technologies, Palo Alto, CA). Finally, DNA sequencing was conducted using the Ion Torrent PGM platform (Life Technologies, Carlsbad, CA).

### Bioinformatics Analysis

The raw single-end reads were assessed and subjected to quality control to obtain a set of clean sequence reads using FastQC and Trimmomatic (Bolger et al., 2014). After pre-processing, FastQC was used again to report features of the pre-processing libraries and verify the effectiveness of trimming. Chimeric sequence removal, operational taxonomic unit (OTU) picking, and taxonomic assignment were performed using Quantitative Insights Into Microbial Ecology (QIIME) v1.9.1 (Caporaso et al., 2010) and Microbiome Helper package (Comeau et al., 2017). In brief, to search for and remove chimeric sequences, we used the script chimera_filter.pl with the database RDP_trainset16_022016.fa, which uses VSEARCH v1.11.1 (Rognes et al., 2016). For OTU classification, we assigned the reads into OTUs at 97% identity using QIIME 1 v1.9.1 with open-reference OTU picking and Greengenes database version 13.8. The output OTU table was pre-processed to remove unwanted OTUs including single and low-confidence OTUs using a dynamic cutoff of <0.1% of the total number of sequences with the script remove_low_confidence_otus. py. Finally, all samples inside the OTU table were subsampled to equal depths using the normalized script single_rarefaction.py (12,600 reads) prior to downstream analysis. No samples were removed in the rarefaction step.

Oligotyping analysis was performed to inspect concealed diversity within OTUs inside the identified genus (Eren et al., 2014). Extracted sequences for oligotyping analysis were assigned by QIIME to the genus *Lactobacillus* with lengths that ranged from only 400 to 409 bp (V4–V5 regions in the 16S rRNA gene). The following parameters were applied: use of 10 entropy components and minimum substantive abundance filter threshold set to 10 reads in all cases. The raw sequencing data of 40 samples was deposited at NCBI Sequence Read Archive database under the accession number PRJNA610712.

### Measurement of Immunoglobulin a Levels

Total concentrations of IgA were quantified in serum and lumen samples. The lumen of the small intestine or colon was flushed with 10 ml PBS and then the content was collected in a 10-ml tube. The content was centrifuged at 500 × g and the supernatant was used. Levels of IgA were measured using the mouse IgA ELISA Ready-SET-Go kit (eBioscience, San Diego, CA, United States). All samples were pre-diluted 10,000-fold before use.

### Intestinal Histopathology

Histopathology analysis of the proximal small intestine, distal small intestine, and colon were performed at particular times post-irradiation (D1, D3, and D10). The tissues were washed with PBS and subsequently fixed with a 4% paraformaldehyde solution. After tissue processing with an ethanol gradient and paraffin-embedding, 4-μm paraffin sections were processed and stained with hematoxylin and eosin (H&E). After staining, sections were dehydrated and mounted with Permount mounting medium (Fisher Scientific, Belgium). Histological scores were determined as follows: epithelial surface erosion, distortion of the villous and crypt architecture, and reduced villous height. The score was graded in a blinded manner as 0 for normal, 1 for mild, and 2 for severe for each parameter. In addition, the intestinal crypt depth was also assessed from the H&E-stained sections and represented as % of the control.

### Crypt Isolation and Organoid Culture

Small intestines were opened longitudinally, cut into 0.5-cm pieces, and washed with ice-cold PBS. Intestinal crypts were isolated by treatment with Gentle Cell Dissociation Reagent (StemCell Technologies, Cambridge, MA) and then passed through a 70-μm cell strainer. Equal numbers of isolated crypts were mixed 1:1 (vol/vol) with Matrigel (BD Biosciences, Franklin Lakes, NJ) and plated in 48-well plates. Matrigel was polymerized by incubating it at 37°C for 10 min, and 400 μl of Intesticult™ OGM Mouse Basal Medium (StemCell Technologies, Cambridge, MA) was added into each well. The culture medium was replaced every 2–3 days and the organoids were passaged by mechanical dissociation every 7 days. To determine the effect of *L. acidophilus*, crypts were cultured in the presence of heat-killed *L. acidophilus*, the formation of organoids was observed at passage 2 using a light microscope and the presence of functional cells was analyzed by immunofluorescence followed by confocal microscopy. Intestinal organoids were harvested using Cell Recovery Solution (Corning, NY). The organoid pellets were resuspended in TRIZOL reagent (Ambion, CA) for RNA isolation.

### Quantitative Reverse Transcriptase-Polymerase Chain Reaction (qPCR) Analysis

RNA was converted to cDNA using reverse transcription reagents (TOYOBO, Japan) according to the manufacturer's protocol. RT-PCR was performed using SYBR Green Real time PCR Master Mix Kit (TOYOBO, Japan) with primers listed in Table S3. The reaction was performed with an ABI StepOne plus real-time PCR machine (Applied Biosystems™, CA) at the Soonchunhyang Biomedical Research Core Facility of Korea Basic Science Institute. Expression of target genes was calculated by comparison of relative levels after normalization to GAPDH.

### Immunofluorescence Staining

Organoids were washed with PBS twice and collected by Cell recovery solution (Corning, NY). Organoids were fixed with 4% paraformaldehyde and then permeabilized with 0.1% tween-20 and 0.2% triton-X100 in PBS. The samples were then incubated with 5% bovine serum albumin for 1 h. Anti-Mucin-2 (Santa Cruz Biotechnology, Santa Cruz, CA) and Alexa Flour 488 conjugated anti-mouse IgG (Life Technologies, Gaithersburg, MD) were used as primary and secondary antibodies, respectively. The organoids were imaged by confocal microscope (LSM 710; Carl Zeiss) at the Soonchunhyang Biomedical Research Core Facility of Korea Basic Science Institute.

### Stool Water Content

Wet stool weight was measured immediately after collection. Stool was dried at 60°C for 24 h, and then, the dry stool weight was measured. The stool water content was calculated by subtracting dry stool weight from the corresponding wet stool weight.

### Statistical Analysis

Statistical analysis of 16S rRNA results was performed using Calypso version 8.72 (Zakrzewski et al., 2017). The data analysis was performed on a rarefied OTU table, and the relative abundance was used for all statistical analyses. To test the statistical significance of alpha diversity estimates among mouse groups at different times, an ANOVA test was applied (Shannon index, Richness, and Chao1). Analysis of beta diversity can measure the between-group differences in diversity. Therefore, principal coordinate analysis (by unweighted UniFrac distance) and supervised multivariate redundancy analysis were performed. Statistical analyses of general observations were conducted using GraphPad software (GraphPad, San Diego, CA, United States) with a Student's *t*-test. Values are presented as the mean ± standard deviation. A *p*-value < 0.05 was considered statistically significant.

## Results

### The Distal Small Intestine Is Most Sensitive to Gamma Irradiation

**Figure 1:**
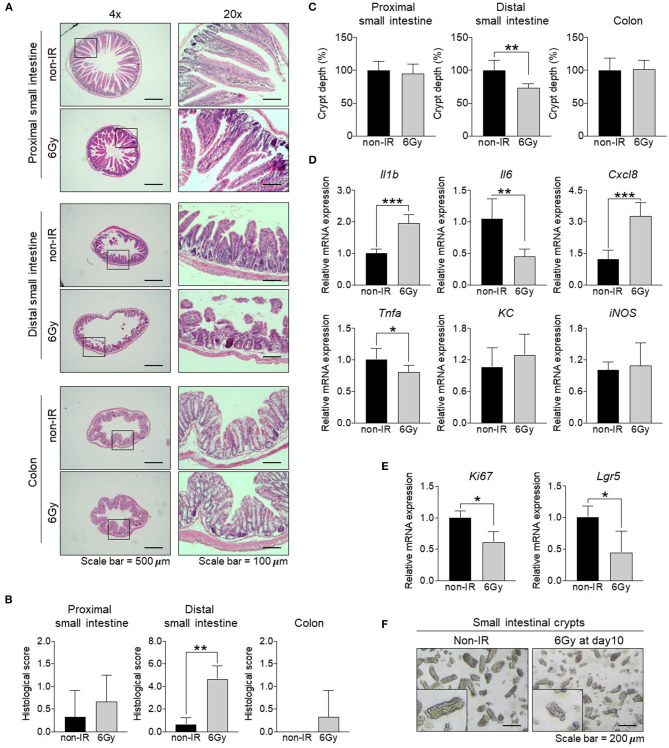
Effect of gamma irradiation on the intestinal tract. Comparison of the small intestine and colon between non-irradiated and irradiated mice at 10 days post-irradiation. **(A)** H&E staining of the proximal and distal small intestine, as well as the colon (magnification, ×40 and ×200). **(B)** Histological scores. **(C)** Assessment of crypt depth (%) measured from the H&E-stained sections. **(D)** The relative mRNA expression of pro-inflammatory cytokines (*Il1b, Il6*, and *Tnfa*), chemokines (*Cxcl8* and *KC*), and *iNOS* in the distal small intestine. **(E)** The relative mRNA expression of a proliferative marker (*Ki67*) and intestinal stem cell marker (*Lgr5*) in the distal small intestine. **(F)** Light microscopy image of an intestinal crypt (magnification, ×100). The data are presented as the mean ± standard deviation, ^*^p < 0.05, ^**^p < 0.005, ^***^p < 0.0005.

To determine the effect of a sublethal dose of gamma irradiation on the intestinal tract. Mice were administered 6 Gy gamma irradiation and alterations to the intestinal tract were investigated 10 days post-irradiation. The body weight of irradiated mice was slightly decreased by ~5% compared to the starting weight. A comparison of non-irradiated and irradiated mice at the same time point revealed that irradiated mice lost ~10% of their body weight (Figure S1A). Stool water content of irradiated mice was slightly increased compared to that in non-irradiated mice (Figure S1B). The length of the small intestine from irradiated mice was shorter compared to that in non-irradiated mice and that of the colon was comparable between the groups (Figure S1C). To analyze the intestinal pathology, the proximal small intestine, distal small intestine, and colon were subjected to H&E staining at 1, 3, and 10 days post-irradiation (D1, D3, and D10). The crypt-villus structure in the proximal small intestine was damaged at D1; however, it was recovered at D3 and D10. Unlike the distal small intestine, the crypt-villus structure was dramatically damaged upon irradiation until D10, compared to that in non-irradiated mice. In contrast, the histopathology results of the colon were comparable between irradiated and non-irradiated mice (Figure 1A, Figure S2). Further, histological scores were evaluated at D10 based on epithelial surface erosion, distortion of the villous and crypt architecture, and reduced villous height (Figure 1B). Histological scores of the distal small intestine from irradiated mice were increased compared with those for non-irradiated mice. Moreover, crypt depth assessment of the H&E-stained sections at D10 showed that crypt depth was not changed significantly in the proximal small intestine and colon; in contrast, crypt depth of the distal small intestine from irradiated mice was significantly decreased compared with that in non-irradiated mice (Figure 1C). Together, our result suggested that the distal small intestine was the most radio-sensitive part of the intestinal tract. Levels of proinflammatory cytokines and chemokines are known to be correlated with tissue damage (Gerassy-Vainberg et al., 2018). Therefore, the RNA was isolated from whole distal small intestinal tissue and the expression levels of tissue damage-related genes (*Il1b, Il6, Tnfa, Cxcl8, KC*, and *iNOS*) were determined (Figure 1D). The expression of *Il1b* and *Cxcl8* were significantly increased and the expression of *Il6* and *Tnfa* were decreased, whereas the expression of *KC* and *iNOS* were comparable between the groups. This result indicates that damage occurred in the distal small intestine after gamma irradiation. A previous study showed that IL-6 signaling plays an important role in regulating small intestinal crypt homeostasis, including the induction of cell proliferation and stemness (Jeffery et al., 2017). Consistently, our data also showed that the distal small intestinal tissue from irradiated mice expressed lower levels of a proliferative marker (*Ki67*) and intestinal stem cell marker (*Lgr5*) compared to levels in small intestinal tissue from non-irradiated mice (Figure 1E). Consistent with the crypt assessment results, crypts containing stem cells and proliferative cells were isolated from mice and the results showed that crypts derived from the distal small intestine of irradiated mice were markedly shorter than those from non-irradiated mice (Figure 1F). These data suggest that gamma irradiation might induce damage to the distal small intestine by targeting intestinal stem cells and cell proliferation.

### Small Intestinal Organoids Derived From Irradiated Mice Show Impaired Budding

**Figure 2:**
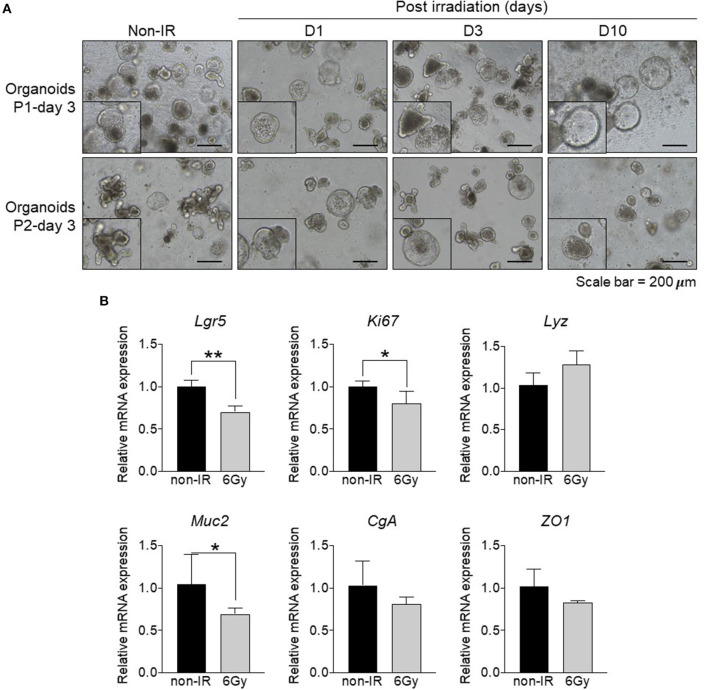
Formation of intestinal organoids derived from distal small intestine of irradiated mice. Comparison between intestinal organoids derived from non-irradiated mice and those from irradiated mice at different post-exposure times (D1, D3, and D10). **(A)** Light microscopy images of intestinal organoids at passage 1 and 2 (magnification, ×100). **(B)** Relative mRNA expression of an intestinal stem cell marker (*Lgr5*), proliferative marker (*Ki67*), Paneth cell marker (*Lyz*), goblet cell marker (*Muc2*), enteroendocrine marker (*CgA*), and tight junction marker (*ZO1*). The data are presented as the mean ± standard deviation, ^*^p < 0.05, ^**^p < 0.005.

Our data clearly showed that the distal small intestine is damaged upon gamma irradiation. To understand the effect of irradiation on intestinal epithelial cells, we utilized the *ex vivo* organoid culture model to study associated phenotypes after gamma irradiation. Intestinal organoid formation is initiated from intestinal crypts, which are composed of ISCs. These cells are located in the crypt region and give rise to progenitor cells that subsequently differentiate into the specialized IECs of the villi. Crypts were isolated from the distal small intestine of non-irradiated (non-IR) or irradiated mice at 1, 3, and 10 days post-irradiation (D1, D3, D10) and cultured to form intestinal organoids for up to 2 passages. The formation of intestinal organoids was observed at passage 1 and passage 2. Organoids derived from non-irradiated mice showed normal budding; however, those derived from irradiated mice showed cystic formation at passage 2 (Figure 2A). These data indicate that gamma irradiation might promote abnormal stem cell functions and cell differentiation processes. Further, the expression of an intestinal stem cell marker (*Lgr5*), proliferative marker (*Ki67*), Paneth cell marker (*Lyz*), goblet cell marker (*Muc2*), enteroendocrine marker (*CgA*), and tight junction marker (*ZO1*) were determined. The expression levels of *Lgr5, Ki67*, and *Muc2* were down-regulated in intestinal organoids derived from irradiated mice (Figure 2B). These data indicate that gamma irradiation alters intestinal stem cell functions, cell proliferation, and differentiation, and especially, goblet cell differentiation.

### Communities of Intestinal Microbiota Are Affected by Gamma Irradiation

**Figure 3:**
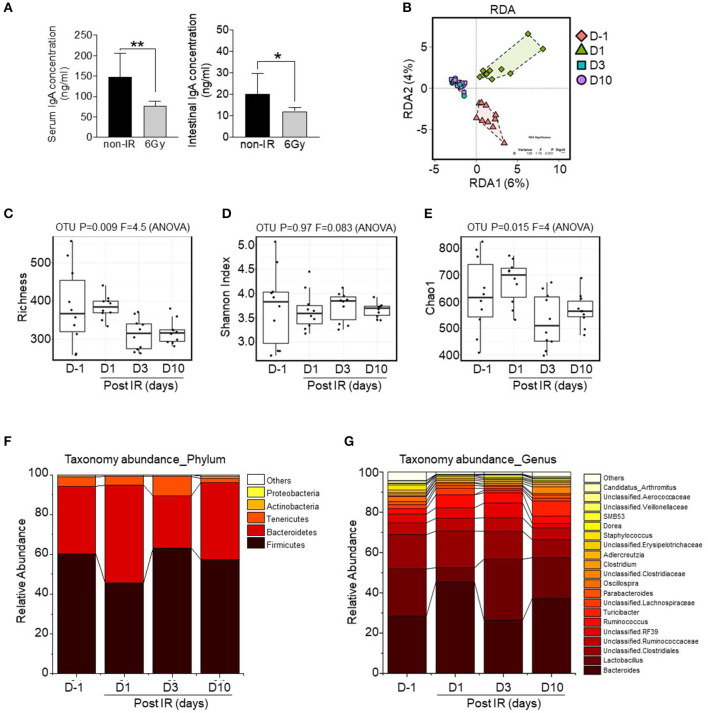
Alterations to the intestinal microbiota composition by gamma irradiation. **(A)** Comparison of IgA levels in serum or intestinal lumens between non-irradiated and irradiated mice. High-throughput sequencing data of 16S rRNA genes were generated from fecal pellets collected from irradiated mice at different time points. **(B)** Beta diversity analysis was performed based on redundancy analysis (RDA). Alpha diversity was also evaluated based on richness analysis **(C)**, Shannon Index analysis **(D)**, and the Chao1 richness method **(E)**. Bar chart of relative abundances of main bacterial phyla **(F)** and bacterial genera **(G)**. The plots are based on the data shown in Table S1. The overall *p*-value was based on ANOVA tests and Wilcoxon rank-sum tests (for pairwise comparisons); ^*^p < 0.05, ^**^p < 0.01.

As levels of immunoglobulin A, the dominant antibody isotype found in mucosal secretions, is associated with alterations to the intestinal microbiome (Nakajima et al., 2018; Catanzaro et al., 2019), we first compared IgA levels in serum and small intestinal lumens between the groups. Results showed that levels in both samples were significantly decreased in irradiated mice (Figure 3A). Therefore, we next aimed to determine changes in the intestinal microbiota following gamma irradiation. Stool samples were collected before irradiation (D−1) and at 1, 3, and 10 days post-irradiation (D1, D3, and D10). After stool DNA extraction, 16S ribosomal RNA (rRNA) sequences were analyzed. The sequencing reads, diversity indices, and sample coverages of the samples included in this study are summarized in Table S1. After quality control processing, a total 814,496 reads (in 40 samples; average of 20,362 sequences per sample) were analyzed for abundance, diversity, and taxonomic comparisons. Based on redundancy analysis, the bacterial communities at different time points were clearly divided into groups (Figure 3B), meaning that there was variation in intestinal microbiota compositions before and at different time points post-irradiation. Operational taxonomic units (OTUs), estimated based on richness analysis, the Chao method, and Shannon indices, revealed that the diversity of microbiota was reduced after irradiation, especially at D3 and D10. Clear differences in diversity were also shown based on OTUs and OTUs estimated by richness analysis (*p* = 0.009; Figure 3C), whereas Shannon indices and the Chao method showed differences of *p* = 0.97 and *p* = 0.015, respectively (Figures 3D,E).

Among bacterial phyla, Firmicutes was sensitive to irradiation, based on the observed reduction in relative abundance at D1; however, it was recovered at later time points (D3 and D10; Figure 3F). Taxonomic abundance analysis at the genus level is shown in Figure 3G. *Bacteroides, Lactobacillus*, and unclassified bacteria were the most abundant genera of all groups. Remarkably, the relative abundance of *Lactobacillus* was dramatically reduced after irradiation at D1, suggesting that this genus is the most radio-sensitive. Additionally, the relative abundance represented by the total sum scaling of bacteria of the top eight genera (*p*-value < 0.001) is shown in Figure S3. Core microbiome analysis revealed the occurrence of irradiation-resistant bacteria such as *Ruminococcaceae, Lachnospiraceae*, and *Clostridiaceae*. These data suggest that a sub-lethal dose of gamma irradiation modulates intestinal microbial communities including the diversity and abundance of radio-sensitive bacteria, and particularly those of the genus *Lactobacillus*.

### Identification of Oligotypes With Differential Abundances in the Genus *Lactobacillus*

Based on our 16S rRNA data, the genus *Lactobacillus* was dramatically decreased by irradiation (D1) but was recovered at later time points (D3 and D10) (Figure 3G); however, most bacteria in *Lactobacillus* were unclassified due to inadequate information regarding taxonomic resolution provided by the Greengenes database. Although several analyses of the intestinal microbiome in mice have been performed, there is no report of the composition of this genus at the species level. Therefore, we conducted oligotyping analysis to inspect concealed diversity within OTUs of the genus *Lactobacillus*.

**Figure 4:**
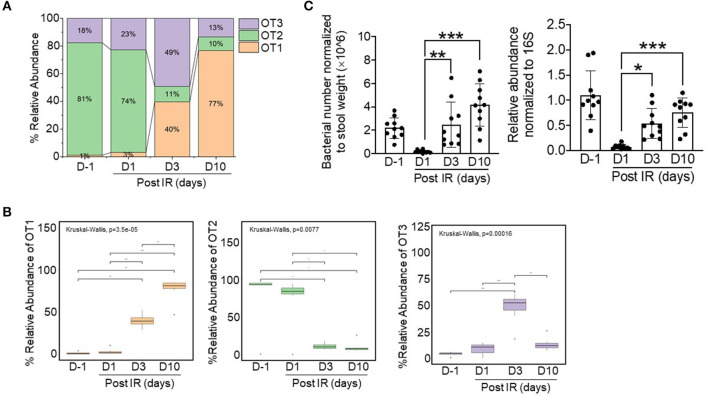
Relative abundances of three dominant oligotypes of the *Lactobacillus* genus across timepoints before (d−1) and after irradiation (d1, d3, and d10). **(A)** Stacked bar plots showing the relative combined abundance of the three oligotypes. **(B)** Boxplot displaying statistical differences across timepoints with respect to specific oligotypes. The boxplot shows the interquartile range (IQR) divided by the median, and the whiskers extend 1.5× the IQR beyond the box. The overall *p*-value was based on Kruskal–Wallis tests and Wilcoxon rank-sum tests (for pairwise comparisons); ^*^p < 0.05, ^**^p < 0.01, ^***^p < 0.001. Bacterial numbers and relative abundances of *L. acidophilus* in stool samples were normalized to stool weight [left, **(C)**] and to 16S sequences [right, **(C)**], respectively.

From 77,444 sequences of this genus (lengths ranging from 400 to 409 bp), based on 80 different OTU identifiers corresponding to specific sequences, we found three dominant oligotypes (OT1, OT2, and OT3) with different assigned species information based on BLASTn analysis results on 16S ribosomal RNA sequences (Bacteria and Archaea) database (Table S2). As shown in Figures 4A,B, bacteria in non-irradiated mice (at D−1) were predominantly of the OT2 oligotype; however, after gamma radiation, the relative abundance of this oligotype was remarkably decreased and the intestinal microbiota composition was dominated by OT1 and OT3. At day 10, the OT1 oligotype became the predominant group (~80% of the relative abundance) in irradiated mice. This result demonstrated that there was a taxonomic shift within the *Lactobacillus* genus between OT1 and OT2 oligotypes across time points. In detail, by comparing the ratios of OT1 and OT2 oligotypes based on statistical analysis, we found that the composition of the intestinal microbiota was significantly shifted from day−1 to day 10 (FDR-corrected *p* < 0.001, Wilcoxon rank-sum test) with an increase in OT1 and a decrease in OT2.

As the intestinal microbiota influences recovery after radiation-induced intestinal damage, our oligotyping analysis indicated that bacteria of *Lactobacillus*-related OT1 might have a beneficial role in this process. Furthermore, we identified that bacteria in the OT1 oligotype were comprised of specific species clusters including *Lactobacillus intestinalis, Lactobacillus kitasatonis, Lactobacillus crispatus, L. acidophilus*, and *Lactobacillus ultunensis*. Among these species, we selected *L. acidophilus* as a representative species because it is known to be a predominant species in the small intestine with a beneficial role in recovery after intestinal injury (Sivieri et al., 2013; Sun et al., 2015). To confirm the presence of *L. acidophilus* in mice, bacterial DNA was isolated from stool samples and its presence was quantified by qPCR (Figure 4C); the results showed that the abundance of *L. acidophilus* was consistent with oligotyping data. Thus, we selected *L. acidophilus* as treatment for *ex vivo* experiments.

### The Indigenous Bacteria *L. acidophilus* Promotes Recovery From Irradiation-Induced Intestinal Damage and the Production of Mucus

**Figure 5:**
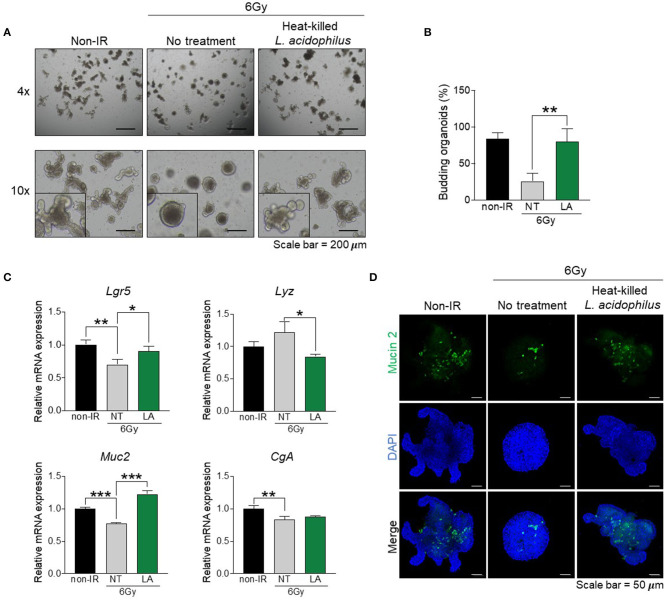
Indigenous *L. acidophilus* protects against irradiation-induced intestinal epithelial cell damage. Intestinal crypts were isolated from the distal small intestine of irradiated mice at 10 days post-exposure. The intestinal crypts were treated with heat-killed *L. acidophilus* and intestinal organoid formation was observed at passage 2. **(A)** Light microscopy images of intestinal organoids (magnification, ×100). **(B)** Proportion of budding organoids. **(C)** Relative mRNA expression of an intestinal stem cell marker (*Lgr5*), Paneth cell marker (*Lyz*), goblet cell marker (*Muc2*), and enteroendocrine marker (*CgA*). **(D)** Immunofluorescence staining of Mucin-2 (magnification, ×100). The data are presented as the mean ± standard deviation, ^*^p < 0.05, ^**^p < 0.005, ^***^p < 0.0005.

Many bacteria reside in the intestinal tract, and especially in the small intestine (Walter, 2008), and these have been shown to improve intestinal dysfunction *in vivo* (Ciorba et al., 2012; Ki et al., 2014; Sun et al., 2015). Our microbiome data showed that bacteria of the genus *Lactobacillus* were dramatically reduced by gamma irradiation (D1) and recovered later post-exposure (D3 and D10) (Figure 3G). In addition, oligotyping data revealed that OT1-related *L. acidophilus* was a representative species (Figures 4A,B). We thus hypothesized that indigenous *L. acidophilus* might play a role in intestinal recovery after gamma irradiation-induced intestinal injury. The isolated crypts were cultured in the presence or absence of heat-killed *L. acidophilus*. Organoid formation and mRNA gene expression were assessed at passage 2. The result showed that treatment with heat-killed *L. acidophilus* could improve the formation and budding of intestinal organoids derived from irradiated mice (Figures 5A,B). In contrast, treatment with heat-killed *L. acidophilus* did not affect the formation and budding of intestinal organoids derived from non-irradiated mice (data not shown). Further, treatment with heat-killed *L. acidophilus* could also reverse the alterations in the *Lgr5* and *Muc2* expression of intestinal organoids derived from irradiated mice (Figure 5C). Further, the recovery of Mucin-2-producing goblet cells was confirmed by immunofluorescent staining as shown in Figure 5D. These data suggest that alterations to IECs mediated by gamma irradiation can be reversed by *L. acidophilus* treatment.

## Discussion

The intestinal tract is a highly complex organ that performs multiple dynamic physiological and biological functions. The intestinal microbiota communicates with the host in a beneficial manner, in addition to impacting host development, health, and pathogenesis. For example, it can promote intestinal epithelial regeneration, barrier integrity, and mucosal immune homeostasis (Parker et al., 2018). In addition, host status also determines the intestinal microbiota composition (David et al., 2014). The intestine is a self-renewing tissue with a high turnover rate throughout life; therefore, it is one of the most susceptible organs to irradiation. Irradiation exposure causes intestinal damage by either directly triggering intestinal stem cell apoptosis (Shadad et al., 2013) or damaging endothelial cell-mediated intestinal stem cell dysfunction and apoptosis (Paris et al., 2001). Previous studies have also shown that the intestinal tract is a radio-sensitive organ (Atasoy et al., 2010; Du et al., 2018; Yu et al., 2018), details of damage to intestinal epithelial cells and how the intestinal microbiota sequentially change following irradiation have not been elucidated. Therefore, it is important to determine the effect of sublethal doses of gamma irradiation on intestinal epithelial characteristics at the cellular level, as well as dynamic changes to the intestinal microbiota community.

Previous studies have demonstrated that exposure to sub-lethal doses of gamma ray induced intestinal damage, including the reduction of crypt depth, villus height, and number of crypts, and persistently delayed intestinal epithelial cell migration in a murine model (Kumar et al., 2018; Venkateswaran et al., 2019). Similar to previous studies, we found that gamma irradiation induces intestinal injury with the loss of crypt–villus architecture, especially in the distal small intestine. Indeed, intestinal organoids derived from irradiated mice show impaired intestinal epithelial stemness and differentiation. The intestinal microbiota is known to be associated with radio-sensitivity (Crawford and Gordon, 2005). Kim et al. previously showed that gamma irradiation alters intestinal microbiota composition in both the small intestine and colon at the genus level (Kim et al., 2015). In this study, we found that bacterial diversity and richness, as well as the ratio of Firmicutes/Bacteroidetes, were reduced by irradiation, which reflects the relative abundance of Firmicutes and an increase in Bacteroidetes, as observed in patients receiving pelvic radiotherapy (Wang et al., 2015). In addition, bacteria of the genus *Lactobacillus* were dramatically reduced by irradiation, suggesting that they are among the most radio-sensitive bacteria. The mechanisms underlying this observation need to be further addressed. Notably, bacteria of *Lactobacillus* were gradually recovered post-irradiation. Our results showed that intestinal damage occurs with the alteration of fecal microbiota composition, especially with reduction in the abundance of *Lactobacillus*. Previous studies have shown that the fecal microbiota of patients with intestinal disease contains lower levels of *Lactobacillus* (Walter, 2008; Carroll et al., 2010). Therefore, the fecal microbiota composition may be associated with the severity of intestinal damage upon irradiation exposure. Moreover, reduction of IgA levels in irradiated mice may also be involved in the alteration of microbiota and intestinal damage due to a lack of proper mucosal protection (Williams and Gibbons, 1972). In addition to *Lactobacillus*, we found that the relative abundance of *Oscillospira* was also consistently reduced by irradiation. This genus comprises slow growing bacteria, and therefore, the association between *Oscillospira* and irradiation is an interesting research avenue for future studies.

The intestinal microbiota plays an important role in intestinal regeneration both under homeostasis conditions and after intestinal damage. Intestinal regeneration is sustained by ISCs; therefore, the intestinal microbiota directly impacts ISC activity in tissue regeneration (Stedman et al., 2016; Lee et al., 2018). Among OT1-related bacteria of the genus *Lactobacillus, L. acidophilus* was reported to be predominant in the small intestine, with a protective role in intestinal injury; furthermore, it is culturable (Sivieri et al., 2013; Sun et al., 2015). Clinical trial studies have shown that *L. acidophilus* or a combination of *L. acidophilus* with *Bifidobacterium bifidum* can preserve intestinal integrity during radiotherapy, represented by reduced radiotherapy-associated diarrhea (Salminen et al., 1988; Chitapanarux et al., 2010; Touchefeu et al., 2014). The decreased mucosal thickness and shortened villous height of the rat small intestine upon irradiation exposure could be recovered by the administration of *L. acidophilus* (Ki et al., 2014). Administration of *Lactobacillus* could also elevate the expression of the intestinal stem cell marker *Lgr5* and increase the number of secretory cells (Lu et al., 2020). In addition, high abundance of *L. acidophilus* or *in vitro* treatment with *L. acidophilus* can protect against tight junction damage and increase the number of goblet cells as well as Mucin-2 expression (Montalto et al., 2004; Zhang et al., 2017). However, the direct evidence and mechanisms underlying its beneficial effects on intestinal epithelial cell recovery are still unknown. In accordance with this, our high-throughput 16S rRNA gene sequencing analysis showed that *L. acidophilus* is gradually increased in the stool long-term following irradiation *in vivo*, suggesting that this species might exert beneficial effects with respect to recovery from irradiation-induced intestinal damage. Interestingly, the components of bacteria are involved in communication with the host. One previous study has reported that *L. acidophilus*-derived molecules can ameliorate intestinal disorders (Sahay et al., 2015). Consistently, we proved that heat-killed *L. acidophilus* can improve budding and can rescue the expression of Mucin-2 in irradiation-damaged intestinal organoids. However, direct evidence showing the beneficial effect of *L. acidophilus* in mitigating radiotoxicity in the small intestine, as well as the mechanisms underlying *L. acidophilus*-mediated amelioration of intestinal cell damage, which occurs partially via the induction of the differentiation of mucin-producing cells, needs to be addressed in further studies.

Taken together, our current study shows that gamma irradiation leads to IEC damage in the distal small intestine and alterations to the intestinal microbiota. Moreover, we show for the first time that the indigenous bacterium *L. acidophilus* has a beneficial role in recovery from irradiation-induced intestinal injury by improving intestinal stem cell function and cell differentiation, at least in part through the induction of mucin-producing cells. Our findings provide a therapeutic perspective of the role of *L. acidophilus* in irradiation-induced intestinal toxicity.

## Data Availability Statement

The datasets presented in this study can be found in online repositories. The names of the repository/repositories and accession number(s) can be found at: https://www.ncbi.nlm.nih.gov/, PRJNA610712.

## Ethics Statement

The animal study was reviewed and approved by Institutional Animal Care and Use Committee at the Korea Atomic Energy Research Institute.

## Author Contributions

PS, J-HS, and YL were responsible for conceptualization of the study and wrote the manuscript. PS performed the experiments and analyzed the data. HP and CP analyzed NGS and bioinformatics. G-UK prepared the *L. acidophilus* culture. YZ, HJ, AJ, and HS performed the irradiation experiments. YL, J-HS, and HS supervised the work. YL was responsible for funding acquisition. All authors contributed to the article and approved the submitted version.

## Conflict of Interest

The authors declare that the research was conducted in the absence of any commercial or financial relationships that could be construed as a potential conflict of interest.
