# Supplementary material for: Irradiation-Induced Intestinal Damage Is Recovered by the Indigenous Gut Bacteria Lactobacillus acidophilus
Source: Front Cell Infect Microbiol. 2020 Aug 18;10:415. doi: 10.3389/fcimb.2020.00415 (PMC7461978; doi:10.3389/fcimb.2020.00415)
Supplement: Supplementary file 1 [file Data_Sheet_1.pdf]

Table S1 Results of sequencing reads, pre-processing reads, and alpha diversity indices of stool samples included in this study

Day of harvest	Sample	Total reads	Analyzed reads	Observed OTUs[a]	Shannon	Simson	Chao1
Day -1	D-1_m1	9,497	3,344	519	4.65	0.95	794.3
	D-1_m2	17,605	10,599	398	4.01	0.90	628.0
	D-1_m3	23,274	12,699	357	4.04	0.94	570.79
	D-1_m4	10,435	6,478	314	2.82	0.73	533.31
	D-1_m5	11,768	8,493	262	2.82	0.82	408.81
	D-1_m6	22,916	12,715	557	5.07	0.97	824.6
	D-1_m7	24,120	14,947	377	3.74	0.90	651.66
	D-1_m8	9,143	6,023	259	2.73	0.82	458.08
	D-1_m9	15,933	11,100	337	3.44	0.88	602.68
	D-1_m10	15,667	8,620	473	3.92	0.85	768.55
Day 1	D+1_m1	17,167	10,077	350	3.18	0.78	728.0
	D+1_m2	15,631	8,349	399	3.78	0.86	713.56
	D+1_m3	21,081	9,923	369	3.67	0.84	532.08
	D+1_m4	18,981	9,228	334	3.54	0.84	566.61
	D+1_m5	30,499	16,477	400	3.35	0.78	760.55
	D+1_m6	21,034	9,276	441	4.45	0.96	716.34
	D+1_m7	22,040	12,127	370	3.64	0.87	686.14
	D+1_m8	19,787	10,118	394	3.48	0.80	772.3
	D+1_m9	22,142	11,685	407	4.12	0.92	665.5
	D+1_m10	25,147	14,302	375	3.27	0.77	600.04
Day 3	D+3_m1	18,788	8,873	367	3.87	0.92	649.25
	D+3_m2	18,232	8,617	305	3.73	0.93	484.41
	D+3_m3	18,121	9,043	274	3.99	0.96	414.62
	D+3_m4	18,678	11,437	279	3.37	0.87	398.0
	D+3_m5	24,952	11,752	338	3.84	0.92	672.0

	D+3_m6	23,077	10,989	371	4.12	0.94	621.62
	D+3_m7	21,794	10,507	326	3.86	0.94	535.39
	D+3_m8	26,671	12,547	266	3.34	0.86	450.53
	D+3_m9	18,964	9,729	341	3.95	0.93	606.0
	D+3_m10	13,750	7,382	263	3.26	0.89	455.45
Day 10	D+10_m1	24,858	11,430	360	3.93	0.92	611.23
	D+10_m2	22,449	11,278	282	3.46	0.89	564.16
	D+10_m3	28,105	14,312	291	3.67	0.91	496.5
	D+10_m4	29,007	15,871	324	3.74	0.93	564.05
	D+10_m5	24,090	12,094	293	3.72	0.93	552.0
	D+10_m6	23,818	11,806	299	3.76	0.91	474.77
	D+10_m7	27,386	12,933	314	3.53	0.86	573.59
	D+10_m8	26,327	16,515	325	3.61	0.90	688.38
	D+10_m9	15,100	8,111	380	3.73	0.87	620.51
	D+10_m10	16,462	9,333	319	3.45	0.85	540.48

[a] The operational taxonomic units (OTUs) were determined based on 97% 16S rRNA gene similarity

Table S2 The three dominant oligotypes with species information based on BLASTn analysis

Oligotype (number of sequences)	Top 5 BLAST species (16S rRNA genes)	NCBI Accession number
OT1	*Lactobacillus intestinalis* strain TH4 16S ribosomal RNA, partial sequence	NR_117071
	Lactobacillus kitasatonis strain JCM 1039 16S ribosomal RNA, partial sequence	NR_024813
	Lactobacillus crispatus strain DSM 20584 16S ribosomal RNA, partial sequence	NR_119274
	Lactobacillus acidophilus strain NBRC 13951 16S ribosomal RNA, partial sequence	NR_113638
	Lactobacillus ultunensis strain CCUG 48460 16S ribosomal RNA, partial sequence	NR_117065
OT2	*Lactobacillus apodemi* strain DSM 16634 16S ribosomal RNA, partial sequence	NR_112752
	Lactobacillus animalis strain KCTC 3501 16S ribosomal RNA, partial sequence	NR_041610
	Lactobacillus murinus strain NBRC 14221 16S ribosomal RNA, partial sequence	NR_112689
	Lactobacillus faecis strain AFL13-2 16S ribosomal RNA, partial sequence	NR_114391
	Lactobacillus salivarius strain HO 66 16S ribosomal RNA, partial sequence	NR_028725
OT3	*Lactobacillus caviae* strain MOZM2 16S ribosomal RNA, partial sequence	NR_157747

	Lactobacillus reuteri DSM 20016 16S ribosomal RNA, partial sequence	NR_075036
	Lactobacillus frumenti strain TMW 1.666 16S ribosomal RNA, partial sequence	NR_025371
	Lactobacillus panis strain DSM 6035 16S ribosomal RNA, partial sequence	NR_026310
	Lactobacillus oris strain DSM 4864 16S ribosomal RNA, partial sequence	NR_026309

Table S3. List of primer sequences for qPCR

Primers	Sequences (5′ – 3′)
16S universal_Forward	ATT ACC GCG GCT GCT GGC
16S universal_Reverse	ATC CCT ACG GGA GGC AGC AGT
Lactobacillus_Forward	TGG AAA CAG GTG CTA ATA CCG
Lactobacillus_Reverse	CCA TTG TGG AAG ATT CCC
*Gapdh*_Forward	TTG ATG GCA ACA ATC TCC AC
*Gapdh*_Reverse	CGT CCC GTA GAC AAA ATG GT
*Ki67*_Forward	CCA GCT GCC TGT AGT GTC AA
*Ki67*_Reverse	TCT TGA GGC TCG CCT TGA TG
*Lgr5*_Forward	ACC CGC CAG TCT CCT ACA TC
*Lgr5*_Reverse	GCA TCT AGG CGC AGG GAT TG
*Zo1*_Forward	GGA GCT ACG CTT GCC ACA CT
*Zo1*_Reverse	GGT CAA TCA GGA CAG AAA CAC AGT
*Muc2*_Forward	ATG CCC ACC TCC TCA AAG AC
*Muc2*_Reverse	GTA GTT TCC GTT GGA ACA GTG AA
*Lyz*_Forward	GAG ACC GAA GCA CCG ACT ATG
*Lyz*_Reverse	CGG TTT TGA CAT TGT GTT CGC
*CgA*_Forward	AAG GTG ATG AAG TGC GTC CT
*CgA*_Reverse	GGT GTC GCA GGA TAG AGA GG
*Il1b*_Forward	GCA ACT GTT CCT GAA CTC AAC
*Il1b*_Reverse	ATC TTT TGG GGT CCG TCA ACT
*Il6*_Forward	TAG TCC TTC CTA CCC CAA TTT CC
*Il6*_Reverse	TTG GTC CTT AGC CAC TCC TTC
*Cxcl8*_Forward	TCA TCA TGC TGT TCT GCT ACG GG
*Cxcl8*_Reverse	TCA GCA TGG TTC TCA TGA GGG TG
*Tnfa*_Forward	ACG GCA TGG ATC TCA AAG AC
*Tnfa*_Reverse	GTG GGT GAG GAG CAC GTA GT
*KC*_Forward	CTG GGA TTC ACC TCA AGA AC
*KC*_Reverse	GAA GCC AGC GTT CAC CAG AC
*iNOS*_Forward	CCA AGC CCT CAC CTA CTT CC
*iNOS*_Reverse	CTC TGA GGG CTG ACA CAA GG
