# Supplementary material for: Irradiation-Induced Intestinal Damage Is Recovered by the Indigenous Gut Bacteria Lactobacillus acidophilus
Source: Front Cell Infect Microbiol. 2020 Aug 18;10:415. doi: 10.3389/fcimb.2020.00415 (PMC7461978; doi:10.3389/fcimb.2020.00415)
Supplement: Supplementary file 2 [file Data_Sheet_2.pdf]

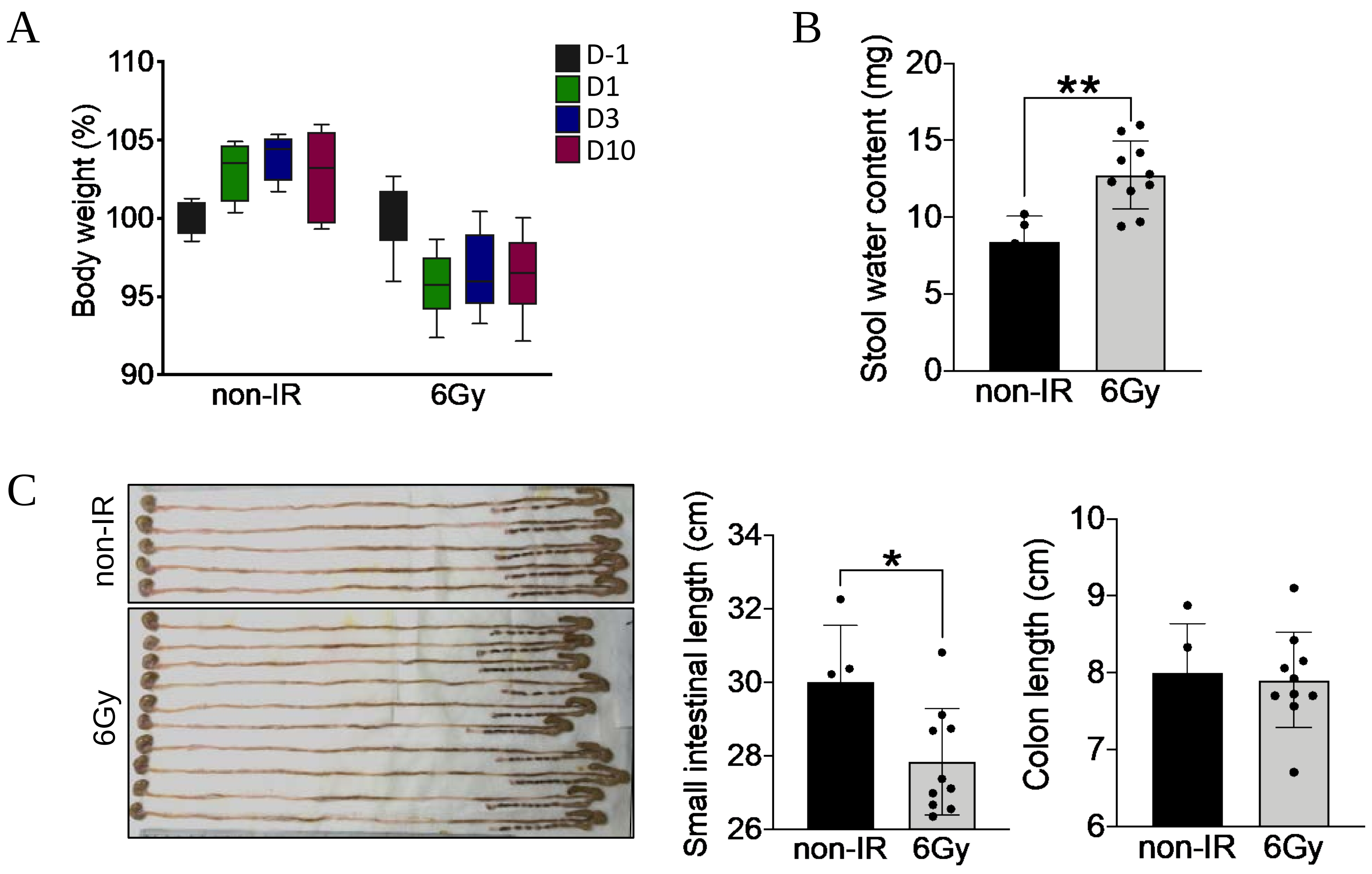

Figure S1 Comparison of different parameters between non-irradiated mice and irradiated mice at D10. **(A)** Percentage of mice body weight. **(B)** Stool water content. **(C)** Length of small intestine and colon. The data are presented as the mean ± standard deviation, *$p < 0.05$, **$p < 0.005$.

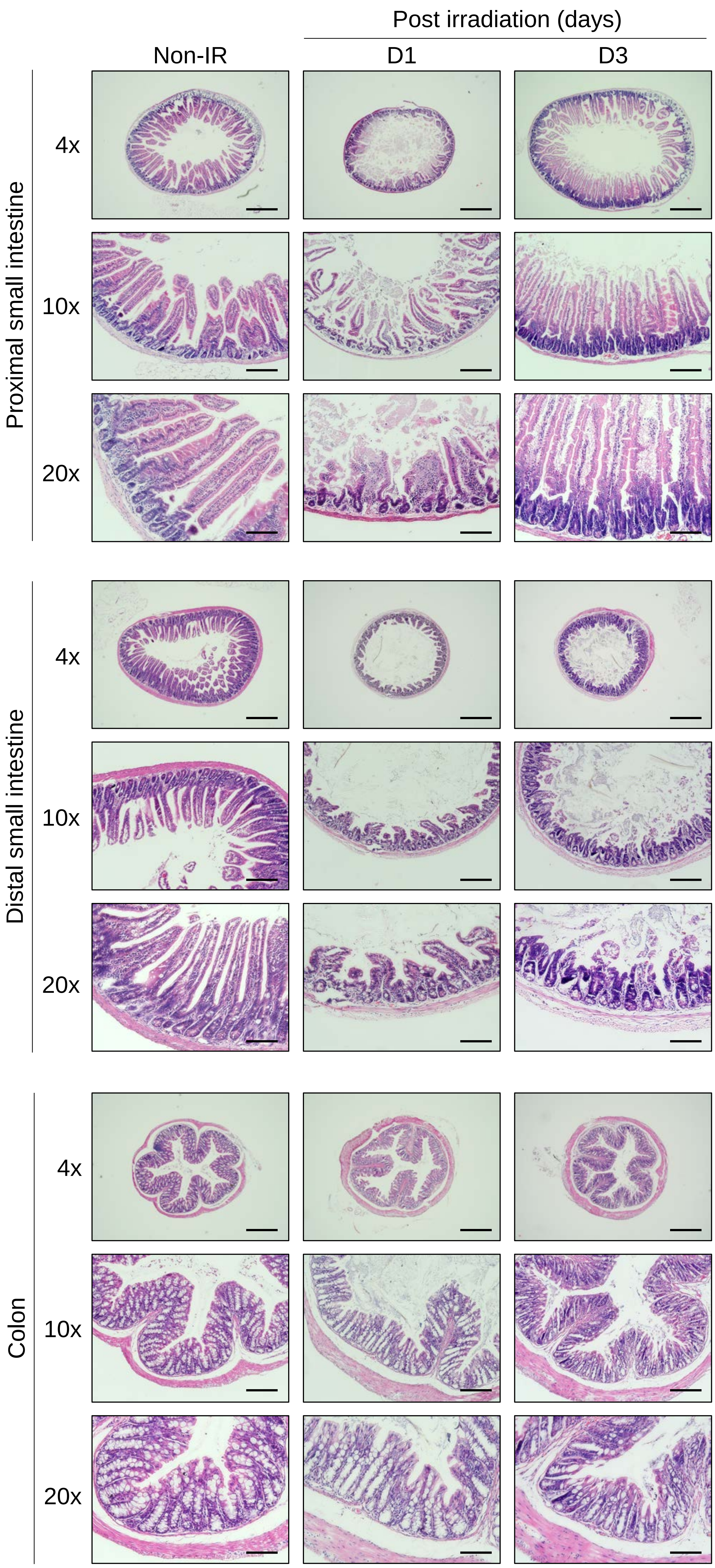

Figure S2 H&E staining of proximal and distal small intestine, as well as the colon (magnification, ×40, ×100 and ×200) at 1 day (D1) and 3 days (D3) post-irradiation compared with non-irradiated mice.

Supplement Figure 2

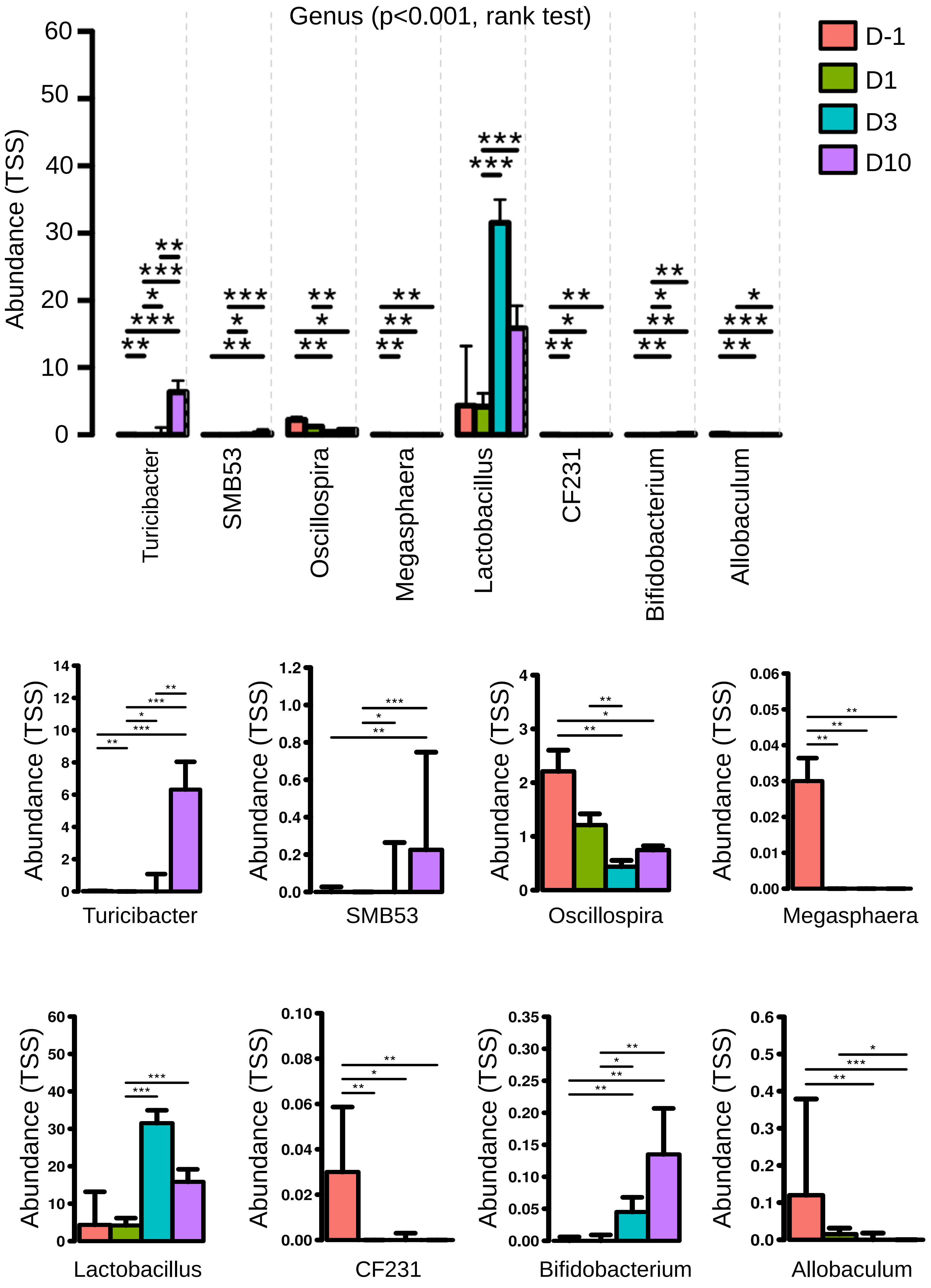

Figure S3 Relative abundance representing total sum scaling (TSS) of bacteria of top eight genera (p-value < 0.001) at different time points (D-1, D1, D3, and D10). The overall *p*-value was based on a Wilcoxon rank-sum test. (*$p < 0.05$, **$p < 0.01$, ***$p < 0.001$).

Supplement Figure 3
